# Genome-Wide Identification and Expression Profile Analysis of the *PHT1* Gene Family in *Gossypium hirsutum* and Its Two Close Relatives of Subgenome Donor Species

**DOI:** 10.3390/ijms21144905

**Published:** 2020-07-11

**Authors:** Sheng Cai, Fujie Liu, Baoliang Zhou

**Affiliations:** State Key Laboratory of Crop Genetics & Germplasm Enhancement, MOE Hybrid Cotton R&D Engineering Research Center, Nanjing Agricultural University, Nanjing 210095, China; 2017201044@njau.edu.cn (S.C.); 2019201060@njau.edu.cn (F.L.)

**Keywords:** phosphate transporter, cotton, phosphorus starvation, phylogenetic analysis, PHT1

## Abstract

Phosphate transporter (PHT) is responsible for plant phosphorus (P) absorption and transport. PHT1 is a component of the high-affinity phosphate transporter system and plays pivotal roles in P absorption under P starvation conditions. However, in cotton, the number and identity of PHT1 genes that are crucial for P absorption from soil remain unclear. Here, genome-wide identification detected twelve PHT1 genes in *Gossypium hirsutum* and seven and eight PHT1 genes in two close relatives of the *G. hirsutum* genome—*G. arboreum* and *G. raimondii*, respectively. In addition, under low-phosphate treatment, the expressions of *GaPHT1;3*, *GaPHT1;4*, and *GaPHT1;5* in roots were upregulated after 3 h of induction, and *GhPHT1;3-At*, *GhPHT1;4-At*, *GhPHT1;5-At*, *GhPHT1;3-Dt*, *GhPHT1;4-Dt*, and *GhPHT1;5-Dt* in the roots began to respond after 1 h of induction. Homologous pairs—*GaPHT1;4* and *GhPHT1;4-At*; *GaPHT1;5* and *GhPHT1;5-At*; *GrPHT1;4* and *GhPHT1;4-Dt*, with *GhPHT1;5-Dt* and *GhPHT1;5-At* being syntenic—were all highly expressed in the roots under normal conditions. Among the genes highly expressed in the roots, *GhPHT1;4-At*, *GhPHT1;5-At*, *GhPHT1;4-Dt* and *GhPHT1;5-Dt* were continuously upregulated by P starvation. Therefore, it is concluded that these four genes might be key genes for P uptake in cotton roots. The results of this study provide insights into the mechanisms of P absorption and transport in cotton.

## 1. Introduction

Phosphorus (P) is an essential macroelement for plant growth and development, playing important roles in plasma membrane maintenance, nucleic acid synthesis, energy metabolism and enzyme regulation [1]. P deficiency strongly affects physiological and biochemical reactions during the growth and development of plants [2,3]. In cotton, P can promote cotton plant growth from the vegetative to reproductive stages, promote cottonseed maturation and boll opening, and increase boll weight [4]. However, under P starvation, the cotton plant exhibits dwarfism, a greatly decreased boll number per plant and stunted development of cotton bolls and cottonseeds [5].

P is plentiful in soil; however, there is very little P available to crops for absorption [1] because most soil P is solidified in an inactive state and cannot be used by plants [6,7]. In China, soil P content is very low and cannot meet the requirements of cotton growth. Therefore, phosphate (Pi) fertilizer must be applied at regular intervals to achieve normal cotton growth [8]. However, excessive use of Pi fertilizer often occurs, which not only depletes Pi resources but also causes water eutrophication and ecological collapse. Therefore, to achieve a balance between environmental protection and the P demand for cotton production, new cotton varieties with high P use efficiency need to be developed.

PHT (phosphate transporter), a member of the major facilitator superfamily (MFS), is located on the plasma membrane. Based on differences in structure and subcellular localization, all Pi transporters from dicotyledons and monocotyledons are divided into four subfamilies, PHT1, PHT2, PHT3 and PHT4, which are mainly located in cell membrane, chloroplast, mitochondria and Golgi apparatus, respectively [9]. PHTs can also be divided into high-affinity (ion concentrations at the micromole level) and low-affinity (ion concentrations at the millimole level) PHT systems. The expression of high-affinity transporters is induced or enhanced in a low-Pi environment. PHT1 belongs to the high-affinity PHT system [10], which is responsible for P absorption by plants under P deficiency conditions [10]. PHT1 consists of 12 hydrophobic transmembrane helical domains. Generally, members of the PHT1 family are highly conserved among species, and their amino acid sequences are highly similar to each other [11].

To date, nine PHT1 genes have been identified in *Arabidopsis thaliana*; these genes have been found to be expressed in P starvation environments. Studies have shown that PHT1 members are expressed in different tissues and organs [12]. *AtPHT1;1* and *AtPHT1;4* play important roles in P absorption at low or high P concentrations [8]. *AtPHT1;5* is very important for Pi distribution from source to sink [13]. *AtPHT1;6* is mainly expressed in floral organs. *AtPHT1;8* and *AtPHT1;9* are strongly induced and expressed in the roots in P deficiency environments and participate in P transport from root to shoot [14]. Thirteen high-affinity Pi transporters have been identified in rice (*Oryza sativa*) [15]. In rice, *OsPT1* is a key participant in P uptake and transport under a high P background [16]. *OsPT2* is a low-affinity P transporter [17]. *OsPT4* is mainly responsible for transporting P from sink to source [18]. *OsPT6* plays extensive roles in P uptake and transport throughout the plant [17]. *OsPT8* is a constitutive transporter [19]. Overexpressing *OsPT9* and *OsPT10* can significantly enhance P uptake [20]. A total of 14 PHT1 genes have been found in soybean [21,22]. In soybean, *GmPT1* and *GmPT2* are expressed in the roots and shoots of seedlings [23]. The expression of *GmPT5* is related to root and nodulation [24]. *GmPT7* is mainly expressed in mature arbuscular mycorrhiza (AM) in the roots [25]. In tomato, five P transporter genes have been identified that belong to the PHT1 family and are mainly expressed in the root hair and root epidermis [26]. In addition, four PHT1 family members have been found in maize [27]. In addition to being studied in the above taxa, the PHT1 family has been studied in alfalfa [28], wheat [29], potato [30], sorghum [31], chrysanthemum [32] and oat [33].

Cotton (*Gossypium* spp.) is one of the most important economic crops, providing the most important natural textile raw material in the world. At present, more than 90% of cultivated cotton is allotetraploid, which arose through the merging of two ancestral genomes 1 or 2 million years ago. *G. arboreum* is a diploid cultivated cotton species, which is a close relative of the A subgenome in cultivated cotton, and *G. raimondii* is the possible donor of the D subgenome [34,35]. Over thousands of years, humans have domesticated wild cotton into cultivated forms that produce fine, spinnable fibers [36]. During domestication, many agriculturally important traits, such as early maturity, seed dormancy, fiber quality (fiber length, fiber strength, and fiber fineness) and yield traits (boll number, boll weight and lint percentage), have been gradually improved. Cotton is one of the most nutrient-demanding crops. However, the phenotypes of human-domesticated cotton do not have a strong ability to absorb nutrient elements; thus, most cultivated cotton species are sensitive to nutrient stress involving P limitation. 

To the best of our knowledge, there are few studies on the molecular mechanisms underlying the responses to low-Pi stress in cotton, and the expression pattern of PHT1 under P starvation has not been fully analyzed [37]. In this study, the most recent genomic and transcriptome data for *G. hirsutum* and genomic and transcriptome data for *G. raimondii* and *G. arboreum* were used to perform genome-wide identification, evolutionary analysis and gene expression analysis of PHT1 family genes. The results provide a basis for the development of P starvation-tolerant cotton.

## 2. Results

### 2.1. PHT1 Genes in G. arboreum, G. raimondii and G. hirsutum

PHT1 is a member of the major facilitator superfamily (MFS). Its main function in plants is to absorb Pi outside the protoplast and maintain the P concentration in the cell. Previous reports have shown that the structure of PHT1 protein in plants is highly conserved and that the protein is localized on the cytomembrane [9]. PHT1 protein contains a conserved domain PF00083 (belonging to the sugar transporter superfamily [38]), and PHT1 genes contain a complete A0109 domain. Based on the conserved domain of PHT1 genes in *A. thaliana*, putative genes of PHT1 in *G. hirsutum*, *G. arboreum* and *G. raimondii* were identified genome-wide (Supplementary Figure S1). Twelve PHT1 genes were identified in *G. hirsutum* (including two sets of tandem duplication genes), seven in *G. arboreum* (including one set of tandem duplication genes), and eight in *G. raimondii*. 

**Figure 1:**
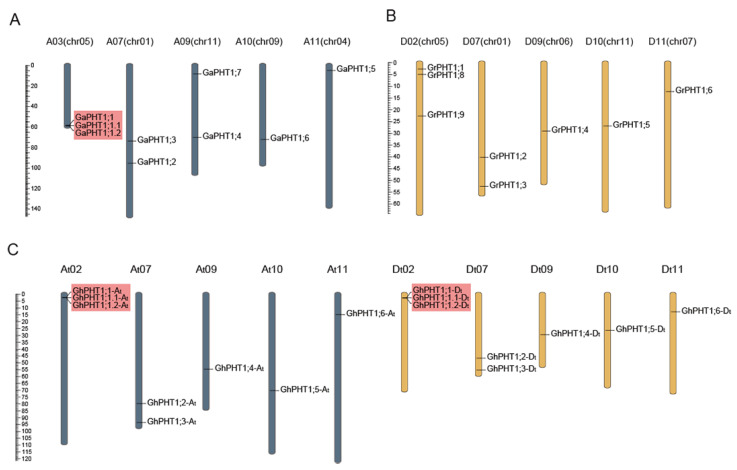
Distribution of phosphate transporter 1(PHT1) genes on chromosomes in *Gossypium*. Scaffolds of *G. arboreum* and *G. raimondii* were reordered. Chromosome codes (A01 to A13, D01 to D13) were assigned based on the collinearity of the interspecific genetic map of allotetraploid cultivated cotton species and the scaffolds (chr01 to chr13) of the *G. arboreum* and *G. raimondii* genomic data. PHT1 genes were named according to the homologous relationship among *Gossypium*, of which *PHT1;1* to *PHT1;6* were homologous genes in the three cotton species, and the remaining without homologous genes were *PHT1;7*, *PHT1;8* or *PHT1;9*; genes with tandem duplication are marked with red boxes. (**A**) *G. arboreum*. (**B**) *G. raimondii*. (**C**) *G. hirsutum.*

The identified PHT1 genes were named according to their homologous relationship, of which *PHT1;1* to *PHT1;6* were homologous genes among the three cotton species, and the remaining without homologous genes were *PHT1;7 PHT1;8,* or *PHT1;9*. Close-linked, tandem duplicate genes were considered a single gene of the gene family. The PHT1 genes in *G. arboreum* were named *GaPHT1;1* to *GaPHT1;7* (Figure 1A); those in *G. raimondii* were named *GrPHT1;1* to *GrPHT1;6*, *GrPHT1;8* and *GrPHT1;9* (Figure 1B); those in *G. hirsutum* were named *GhPHT1;1-At* to *GhPHT1;6-At* and *GhPHT1;1-Dt* to *GhPHT1;6-Dt* (Figure 1C). The PHT1 gene distribution on the chromosomes is shown in Figure 1. The PHT1 genes were distributed on several chromosomes in *Gossypium*. In Table 1, the amino acid (aa) numbers, molecular weights and isoelectric points (pIs) of the PHT1 genes in *Gossypium* are listed. The number of amino acids encoded by PHT1 genes ranged from 513 to 540. The molecular weights ranged from 56.74 to 59.23 kDa, and the pIs ranged from 8.07 to 9.29.

**Figure 2:**
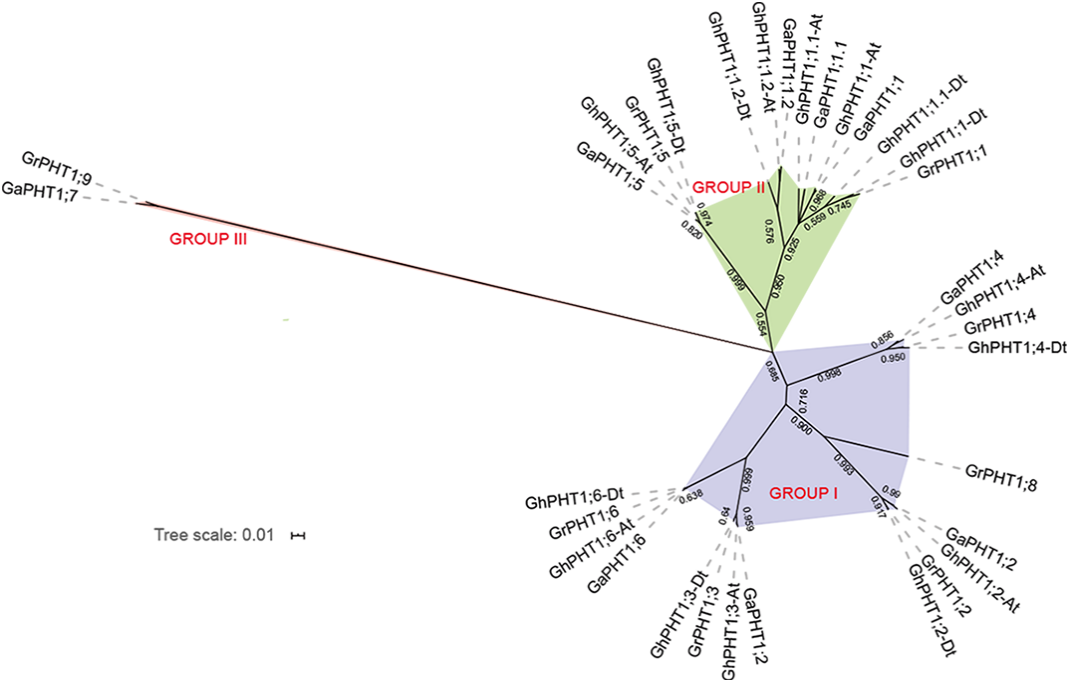
The phylogenetic tree of PHT1 genes in *Gossypium*. Ga, Gr and Gh represent PHT1 genes from *G. arboreum*, *G. raimondii* and *G. hirsutum*, respectively. Different subgroups are marked by different colors and are named Group I, Group II, and Group III.

To investigate the phylogenetic relationships among PHT1 genes in *Gossypium*, we employed the maximum likelihood (ML) method to construct a phylogenetic tree based on the PHT1 protein sequences of *G. arboreum*, *G. raimondii* and *G. hirsutum*. The PHT1 genes were clustered into three subgroups—Group I, Group II and Group III (Figure 2). Group I contained four genes from *G. arboreum*, five from *G. raimondii* and eight from *G. hirsutum*. Group II contained two genes from each of *G. arboreum* and *G. raimondii* and four genes from *G. hirsutum*. Group III contained only one gene from each of *G. arboreum* and *G. raimondii* and no gene from *G. hirsutum*. The phylogenetic tree demonstrated that each gene from *G. arboreum* was mostly closely related to one from *G. hirsutum*, yielding the following pairs: *GaPHT1;1* and *GhPHT1;1-At*; *GaPHT1;1.1* and *GhPHT1;1.1-At*; *GaPHT1;1.2* and *GhPHT1;1.2-At*; *GaPHT1;2* and *GhPHT1;2-At*; *GaPHT1;3* and *GhPHT1;3-At*; *GaPHT1;4* and *GhPHT1;4-At*; *GaPHT1;5* and *GhPHT1;5-At*; *GaPHT1;6* and *GhPHT1;6-At*. Similarly, genes from *G. raimondii* were most closely related to ones in *G. hirsutum*, yielding the following pairs: *GrPHT1;1* and *GhPHT1;1-Dt*; *GrPHT1;2* and *GhPHT1;2-Dt*; *GrPHT1;3* and *GhPHT1;3-Dt*; *GrPHT1;4* and *GhPHT1;4-Dt*; *GrPHT1;5* and *GhPHT1;5-Dt*; *GrPHT1;6* and *GhPHT1;6-Dt*. The members of each of the above pairs of genes were grouped in the same branch. The results revealed homologous relationships between both *GaPHT1* and *GhPHT1* of the A subgenome of *G. hirsutum* and between *GrPHT1* and *GhPHT1* of the D subgenome of *G. hirsutum*. In addition, the pair of genes *GaPHT1;7* (from *G. arboreum*) and *GrPHT1;9* (from *G. raimondii*) clustered together on the same branch, implying that their protein sequences were very different from those of *G. hirsutum*. 

### 2.2. Structural Analysis of PHT1 Genes

**Figure 3:**
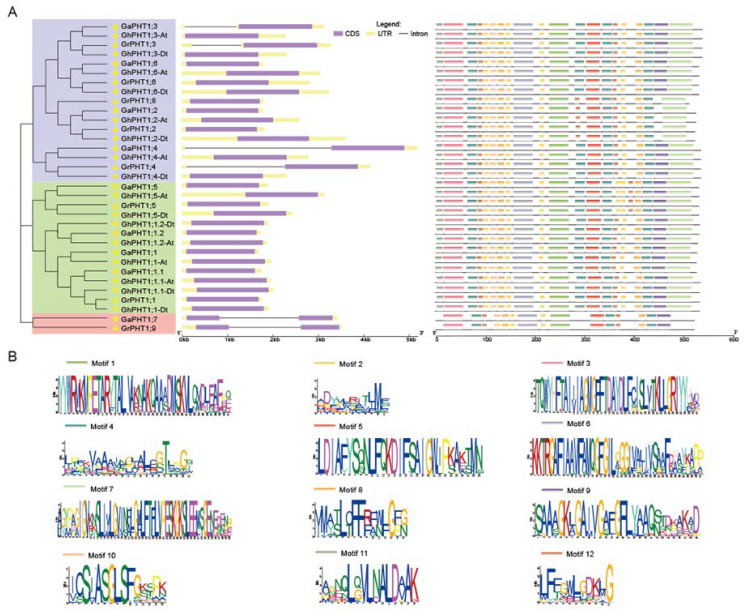
PHT1 gene structure and conserved motifs in *Gossypium*. (**A**) Phylogenetic relationships (left), gene structure (middle), and conserved motifs (right) among PHT1 genes of *Gossypium*. Pentagrams, triangles and circles represent *G. arboreum*, *G. raimondii*, and *G. hirsutum*, respectively. (**B**) Conserved motif sequences of *Gossypium*.

To further understand the gene structure variation of the PHT1 family in cotton, the exons and introns were analyzed by Gene Structure Display Server (GSDS). The results showed that the PHT1 genes in cotton are highly conserved in structure. Genes that were clustered into the same subgroup in the phylogenetic tree were structurally similar. In general, PHT1 genes in cotton contained one or two exons and one or zero introns. There was only one exon in members of Group I and Group II, among which, four genes (*GaPHT1;3*, *GaPHT1;4*, *GrPHT1;3* and *GrPHT1;4*) contained one intron and the remaining 20 genes had no introns. Only two genes in Group III contained two exons and one intron (Figure 3A). The PHT1 gene structure of *Gossypium* was found to be similar to those of *A. thaliana* and *Oryza sativa* [15,39]. This finding indicated that the PHT1 family is highly conserved in both monocotyledons and dicotyledons, implying that the PHT1 genes are very important to plants.

A protein’s motif is closely related to its function. The conserved motifs of PHT1 proteins in cotton were analyzed by MEME software. High similarity in motif structure was observed within a subgroup in the phylogenetic tree. As shown in Figure 3A,B, in genes in both Groups I and II, the protein N-terminal was motif 11, and the protein C-terminal was motif 7. In Group I, five genes (*GaPHT1;2*, *GrPHT1;2*, *GrPHT1;8*, *GhPHT1;2-At* and *GhPHT1;2-Dt*) were very similar in motif structure, having no motif 4 or motif 9, but having an extra motif 12; these genes were different in motif structure from the others. In Group II, four genes (*GaPHT1;5*, *GrPHT1;5*, *GhPHT1;5-At* and *GhPHT1;5-Dt*) had similar structures, with one more motif 2 than the others. Interestingly, the motif structures of genes in Group III differed from those of genes in Groups I and II. For genes in Group III, the protein N-terminal was motif 3 without motif 11. Its C-terminal was motif 9 without motif 7. Compared with other members of the PHT1 family, *GaPHT1;7* and *GrPHT1;9* are both missing two motif 2s and one motif 4. These different motif components might lead to different gene functions.

The prediction of cis-acting elements in the promoter showed that the CAAT-BOX (GGGTCAATCT) and TATA-BOX (TATAATAAT) elements were present in all gene promoters. Other elements were mostly related to light responses and plant hormone responses. The W-box (TTGACY) element was predicted in the promoters of *GaPHT1;4*, *GaPHT1;5*, *GhPHT1;4-At*, *GhPHT1;5-At*, *GhPHT1;4-Dt*, *GrPHT1;2* and *GrPHT1;4*. The PHO-like element (GDHGTGG) was found in *GrPHT1;4* and *GrPHT1;9*. *GhPHT1;3-At*, *GhPHT1;3-Dt* and *GrPHT1;3* contained the root motif box element (ATATT), which is related to root expression (Supplementary Table S1).

### 2.3. Expansion of PHT1 Family Genes in Gossypium

The nuclear genome sizes and gene contents vary greatly in higher plants, mostly due to gene duplication. Gene duplication events can be divided into tandem duplication, segmental duplication/WGD (Whole Genome Duplication), and dispersed duplication events [40]. By analyzing the phylogenetic tree of PHT1 genes in *Gossypium*, *A. thaliana* and rice, it was found that *GaPHT1;1* and *GaPHT1;5* (as well as *GrPHT1;1* and *GrPHT1;5*) originated from the same hypothetical ancestor gene, and their duplication events occurred before the divergence of *G. arboreum* and *G. raimondii* (Figure S2). *GaPHT1;3* and *GaPHT1;6* (as well as *GrPHT1;3* and *GrPHT1;6*) had a common origin, and their duplication events occurred in the ancestral species of *G. arboreum* and *G. raimondii*. *GaPHT1;2* and *GrPHT1;2* were homologous genes, which were derived from the same hypothetical ancestral gene. Similarly, *GaPHT1;4* and *GrPHT1;4* originated from another gene within the ancestor of *G. arboreum* and *G. raimondii*. Interestingly, there were two tandem genes of *GaPHT1;1* but not in *GrPHT1;1*; it is inferred that the tandem duplication event of *GaPHT1;1* occurred after the divergence of *G. arboreum* and *G. raimondii*.

**Figure 4:**
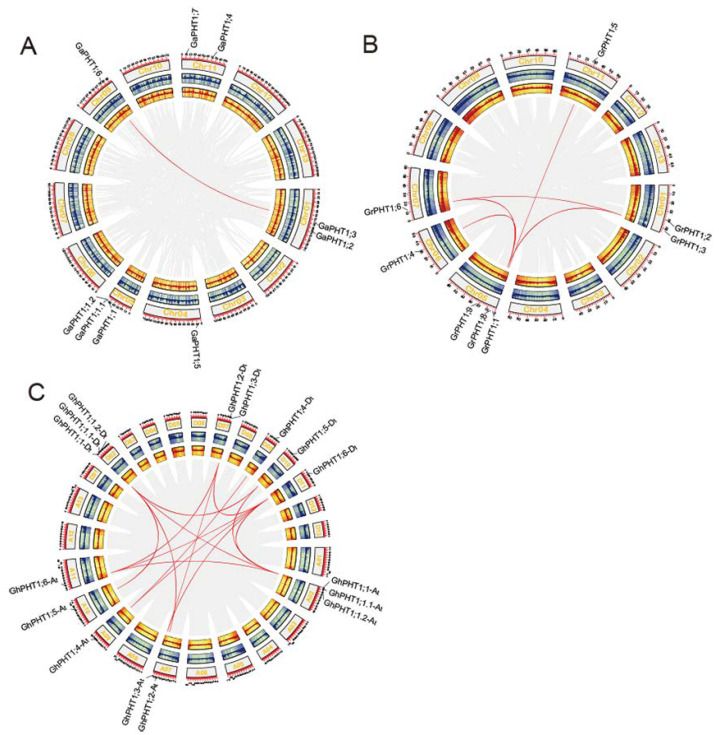
The syntenic relationships of PHT1 genes based on analysis of genome-wide protein sequences using MCScanX [41]. Syntenic relationships with scores < 250 were filtered out. Circos [42] panels from outside to inside represent the following: chromosome, gene density in sense strand and gene density in antisense strand. (**A**) *G. arboreum*. (**B**) *G. raimondii*. (**C**) *G. hirsutum.*

**Figure 5:**
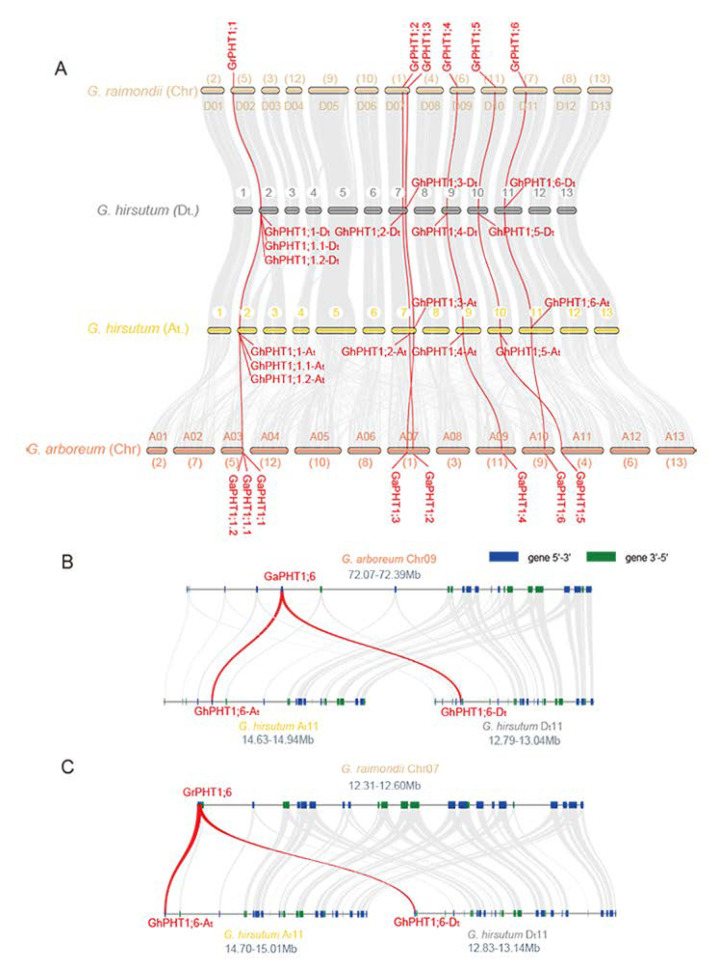
Syntenic relationships among members of the PHT1 family in *Gossypium*. (**A**) Syntenic characterization of *G. arboreum*, *G. raimondii*, and *G. hirsutum*. Scaffolds of *G. arboreum* and *G. raimondii* were reordered. The chromosome codes (A01 to A13, D01 to D13) were assigned based on the collinearity of the interspecific genetic map of allotetraploid cultivated cotton species and the scaffolds (chr1 to chr13) of the *G. arboreum* and *G. raimondii* genomic data. Macrosynteny connecting blocks with greater than 30 gene pairs are represented by grey links. The red line indicates a syntenic relationship between two PHT1 genes. (**B**) Microsyntenic relationships of *G. arboreum* Chr. A11 (chr9), Chr. A_t_11 (A subgenome of allotetraploid cotton) and Chr. D_t_11 (D subgenome). (**C**) Microsyntenic relationships of *G. raimondii* Chr. D11 (chr7), Chr. A_t_11 (A subgenome of allotetraploid cotton) and Chr. D_t_11 (D subgenome). The blue and green boxes indicate the different directions of transcribed genes. Only syntenic relationships with scores ≥ 0.70 are shown.

To further investigate the syntenic relationships of cotton PHT1, we used MCScanX to perform synteny analysis within family members. A pair of syntenic genes, *GaPHT1;3* and *GaPHT1;6*, was found in *G. arboreum* (Figure 4A). Five pairs of syntenic genes (*GrPHT1;1* and *GrPHT1;5*; *GrPHT1;3* and *GrPHT1;6*; *GrPHT1;3* and *GrPHT1;8*; *GrPHT1;4* and *GrPHT1;9*; *GrPHT1;6* and *GrPHT1;8*) were found in *G. raimondii* (Figure 4B). Twelve pairs of syntenic genes (*GhPHT1;1-At* and *GhPHT1;6-At*; *GhPHT1;1-At* and *GhPHT1;1-Dt*; *GhPHT1;1-At* and *GhPHT1;5-Dt*; *GhPHT1;1-At* and *GhPHT1;6-Dt*; *GhPHT1;2-At* and *GhPHT1;1-Dt*; *GhPHT1;3-At* and *GhPHT1;3-Dt*; *GhPHT1;3-At* and *GhPHT1;6-Dt*; *GhPHT1;4-At* and *GhPHT1;4-Dt*; *GhPHT1;4-At* and *GhPHT1;6-Dt*; *GhPHT1;5-At* and *GhPHT1;1-Dt*; *GhPHT1;6-At* and *GhPHT1;3-Dt*; *GhPHT1;6-At* and *GhPHT1;6-Dt*) were found in *G. hirsutum* (Figure 4C). The synteny of PHT1 families was analyzed by the python version of MCScan2. The results revealed syntenic relationships both between *GaPHT1* and the *GhPHT1* of the A subgenome and between *GrPHT1* and the *GhPHT1* of the D subgenome (Figure 5A), whereas *GrPHT1;8* had no syntenic gene in *G. hirsutum* (score ≥ 0.70). *GaPHT1;7* and *GrPHT1;9* each exhibited synteny with the same gene, *GH_D02G1054*, in *G. hirsutum*, but this gene is not in the PHT1 family. Syntenic analysis showed the *GaPHT1;6* had synteny with both *GhPHT1;6-At* (A subgenome) and *GhPHT1;6-Dt* (D subgenome) (Figure 5B) and that *GrPHT1;6* had synteny with these same two genes (Figure 5C). These findings indicate that *GaPHT1;6*, *GrPHT1;6*, *GhPHT1;6-At* and *GhPHT1;6-Dt* are the most conserved genes in the PHT1 family of *Gossypium*. All *GhPHT1* genes have their homologous genes found in the two closely related donor species, so it is inferred that *GhPHT1* genes were obtained from the two donor species.

Therefore, we concluded that most of the PHT1 genes in *G. arboreum* and *G. raimondii* had experienced gene duplication events before these two species diverged. The *GhPHT1* genes were derived from the donor species of subgenomes A and D.

To explore the selection pressure on PHT1 genes after gene duplication, we calculated and analyzed the ratio of the non-synonymous substitution rate to the synonymous substitution rate (Ka/Ks) of homologous PHT1 genes in *Gossypium*. Fourteen pairs of PHT1 genes showing homology between *G. arboreum* and *G. hirsutum* were obtained by bidirectional Blastp: *GaPHT1;1* and *GhPHT1;1-At*; *GaPHT1;1.1* and *GhPHT1;1.1-At*; *GaPHT1;1.2* and *GhPHT1;1.2-At*; *GaPHT1;2* and *GhPHT1;2-At*; *GaPHT1;3* and *GhPHT1;3-At*; *GaPHT1;4* and *GhPHT1;4-At*; *GaPHT1;5* and *GhPHT1;5-At*; *GaPHT1;6* and *GhPHT1;6-At*. Their Ka/Ks values ranged from 0.0867 to 0.3665, with an average of 0.2884. These values are much less than 1, suggesting these genes underwent purifying selection. Six pairs of genes were identified as homologous between *GrPHT1* and *GhPHT1*, namely, *GrPHT1;1* and *GhPHT1;1-Dt*; *GrPHT1;2* and *GhPHT1;2-Dt*; *GrPHT1;3* and *GhPHT1;3-Dt*; *GrPHT1;4* and *GhPHT1;4-Dt*; *GrPHT1;5* and *GhPHT1;5-Dt, GrPHT1;6* and *GhPHT1;6-Dt*. Except for one pair of homologous genes (*GrPHT1;4* and *GhPHT1;4-Dt*), the Ka/Ks values ranged from 0.1149 to 0.8520, with an average of 0.3832, indicating purifying selection. Generally, the Ka/Ks values of homologous *GrPHT1* and *GhPHT1* genes were higher than those of homologous *GaPHT1* and *GhPHT1* genes, implying faster evolutionary rates of *GhPHT1* genes on D_t_ than on A_t_ in *G. hirsutum*. The Ka/Ks value of the pair *GrPHT1;4* and *GhPHT1;4-Dt* was greater than 1, which suggested that these genes have undergone positive selection. Their non-synonymous mutations were retained, which might endow new functions. The specific functions of these genes need to be determined (Table 2).

### 2.4. Expression Patterns of PHT1 Genes in Gossypium

**Figure 6:**
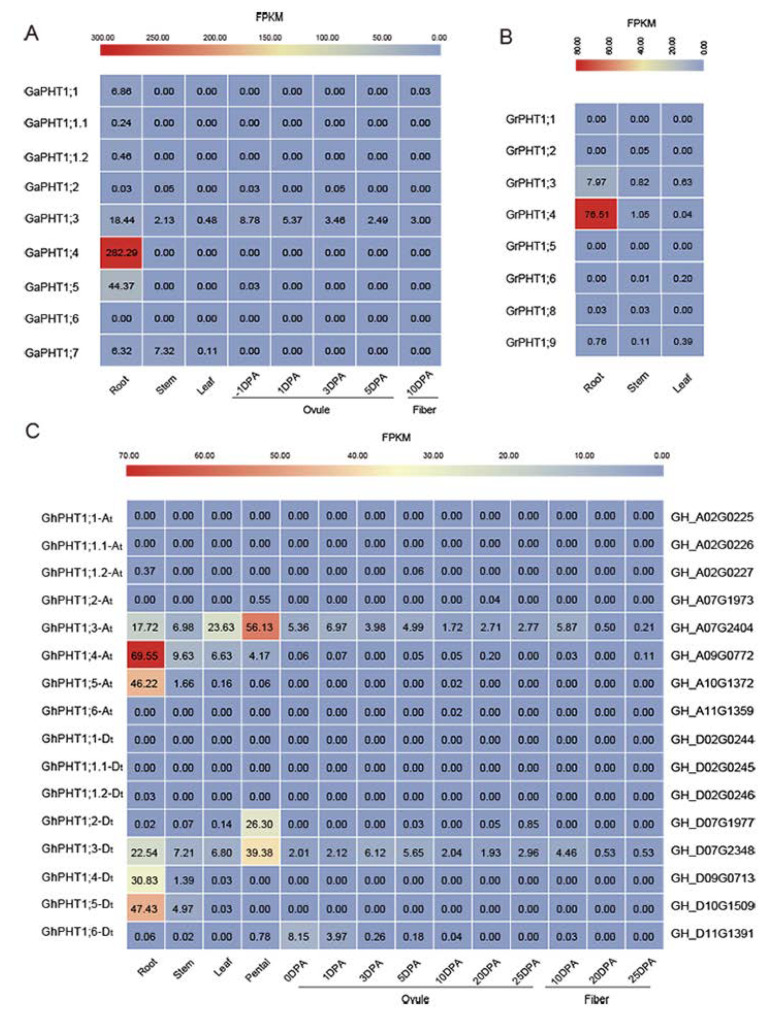
Expression patterns of PHT1 genes in *Gossypium*. Comparative analysis of the transcriptome in different tissues. The expression level of all genes is shown as a colored square at the top of Fragments Per Kilobase per Million (FPKM), indicating a gradual change from minimum (blue) to maximum (red). The value in the square is the FPKM. (**A**) *G. arboreum*. (**B**) *G. raimondii*. (**C**) *G. hirsutum.*

To explore the temporal and spatial expression patterns of PHT1 genes in different tissues of *Gossypium*, transcriptome data were analyzed. The results showed that *GaPHT1;4* and *GaPHT1;5* were strongly expressed in the roots (Figure 6A) and that *GaPHT1;3* was expressed at low levels in root, stem, leaf, ovule and fiber tissues. *GrPHT1;4* was highly expressed in the roots, and *GrPHT1;3* was expressed in the roots at a low level (Figure 6B). In *G. hirsutum*, *GhPHT1;3-At*, *GhPHT1;4-At*, *GhPHT1;5-At*, *GhPHT1;3-Dt*, *GhPHT1;4-Dt* and *GhPHT1;5-Dt* were highly expressed in the roots and stem (Figure 6C), implying that they play roles in Pi absorption [22] and Pi transport [26,28]. *GhPHT1;3-At*, *GhPHT1;4-At* and *GhPHT1;3-Dt* possibly participate in Pi distribution or metabolism in the leaves [13,43]. The expression of *GhPHT1;3-At*, *GhPHT1;4-At*, *GhPHT1;2-Dt* and *GhPHT1;3-Dt* was detected in petals, implying that these genes might function in reproductive growth. *GhPHT1;6-Dt* was expressed in the ovule at 0 and 1 day post anthesis. *GhPHT1;3-At* and *GhPHT1;3-Dt* were expressed in almost all the tissues analyzed. According to the transcriptome data, PHT1 genes in *Gossypium* are mainly expressed in the roots and less in leaf and other tissues; P uptake is mainly dependent on the roots [44]. 

### 2.5. Expression of PHT1 Genes Induced by Low-Pi Treatment

To investigate the potential functions of PHT1 genes under P starvation, seedlings of *G. arboreum* and *G. hirsutum* were treated with low P in a hydroponic environment. The expression of PHT1 genes in the roots and leaves was obtained at 0, 1, 3, 6, 12 and 24 h after induction.

**Figure 7:**
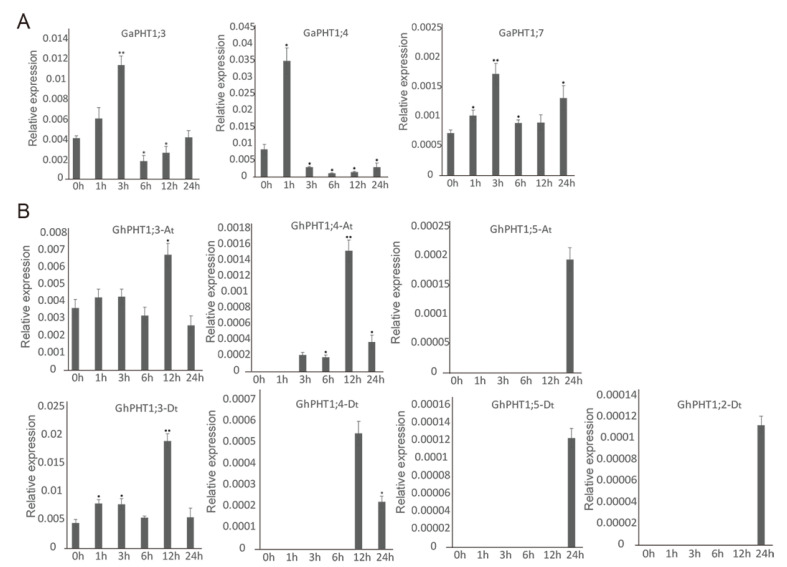
Expression of PHT1 genes determined by qRT-PCR in leaves under phosphorus starvation stress. In the graphs, 0, 1, 3, 6, 12 and 24 h represent the time of low-Pi treatment. The error bars represent the standard deviations of three biological repeats. Statistical analyses were performed by comparing expression levels to that at 0 h without low-Pi treatment using Student’s t-test (* *p* < 0.05, ** *p* < 0.01) [45]. (**A**) *G. arboreum*; (**B**) *G. hirsutum*.

**Figure 8:**
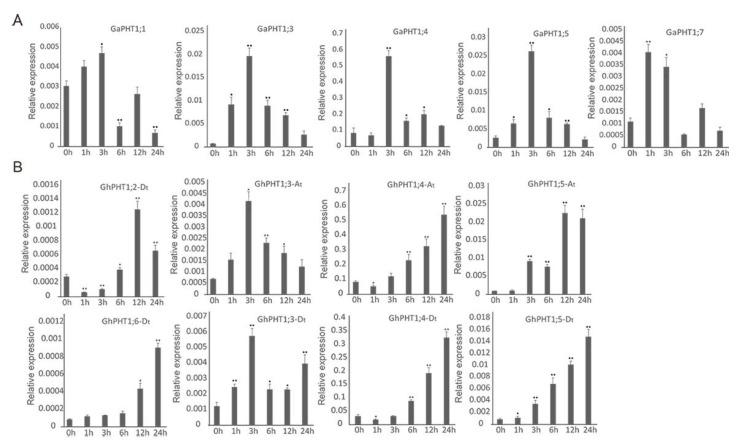
Expression of PHT1 genes determined by qRT-PCR in roots under phosphorus-starvation stress. In the graphs, 0, 1, 3, 6, 12 and 24 h represent the time of low-Pi treatment. The error bars represent the standard deviations of three biological repeats. Statistical analyses were performed by comparing expression levels to that at 0 h without low-Pi treatment using Student’s t-test (* *p* < 0.05, ** *p* < 0.01) [45]. (**A**) *G. arboreum*; (**B**) *G. hirsutum*.

The results indicated that three *GaPHT1* genes were induced in leaf by low-Pi treatment. Relative to its expression in control plants (at 0 h), the expression of *GaPHT1;3* was significantly increased at 3 h and significantly decreased at 6 h. *GaPHT1;4* was significantly upregulated at 1 h. *GaPHT1;7* was significantly upregulated at 1 and 3 h (Figure 7A). In the roots, *GaPHT1;1* was significantly upregulated at 3 h. *GaPHT1;3* and *GaPHT1;5* were significantly upregulated at 1 and 3 h; their expression gradually decreased after 6 h, remaining higher than that at 0 h. *GaPHT1;4* was significantly upregulated at 3 h. *GaPHT1;7* was significantly upregulated at 1 to 3 h (Figure 8A). In general, the upregulation of gene expression induced by low P mainly occurred at 1 to 3 h after induction, while the expression at 6 h was lower than that at 3 h. These results indicated that the main response time of PHT1 genes to low-Pi stress in *G. arboreum* was approximately 1 to 3 h after treatment (Supplementary Table S2).

In *G. hirsutum*, seven PHT1 genes in the leaves responded to low P. *GhPHT1;3-At* was significantly upregulated at 12 h. *GhPHT1;4-At* was expressed at 3 h and significantly upregulated at 12 h. *GhPHT1;3-Dt* was significantly upregulated at 1, 3 and 12 h. The expression of *GhPHT1;4-Dt* was detected at 12 and 24 h. Expression of *GhPHT1;5-At*, *GhPHT1;2-Dt* and *GhPHT1;5-Dt* was not detected until 24 h, occurring at very low levels at this time. No expression of the other five PHT1 genes was detected in the leaves (Figure 7B). In the roots, *GhPHT1;3-At* and *GhPHT1;3-Dt* were significantly upregulated at 3 h. *GhPHT1;4-At* and *GhPHT1;4-Dt* were downregulated at 1 h and significantly upregulated from 6 h after induction. *GhPHT1;5-At* and *GhPHT1;5-Dt* were significantly upregulated from 3 to 24 h. *GhPHT1;2-Dt* was downregulated at 1 h and significantly upregulated at 6 and 12 h. *GhPHT1;6-Dt* was significantly upregulated at 12 and 24 h (Figure 8B). PHT1 genes in the leaves were significantly upregulated at 12 h, indicating that the response of PHT1 genes in the leaves to low-Pi stress was slow. In contrast, the expression of PHT1 genes in the roots was significantly changed at 1 h after induction, indicating that the PHT1 genes in the roots respond quickly to P starvation (Supplementary Table S3).

## 3. Discussion

### 3.1. Homologues PHT1 Genes in G. arboreum, G. raimondii and G. hirsutum 

The Pi transporter PHT1 is highly conserved in plants and has conserved domains in both dicotyledons and monocotyledons; thus, it is amenable to phylogenetic analysis. Previous studies have identified nine PHT1 genes in *A. thaliana* (diploid) [39], 13 in *O. sativa* (diploid) [15] and 14 in *Glycine max* (diploid) [22]. Compared with the *G. hirsutum* genome, the genomes of the above species are smaller but contain similar numbers of PHT1 genes. The phylogenetic analysis revealed the sequence changes of the PHT1 family in *Gossypium* during domestication. In our study, seven, eight and twelve PHT1 genes were found in *G. arboreum*, *G. raimondii* and *G. hirsutum*, respectively. In *G. hirsutum*, six genes each were distributed on the A and D subgenomes, and the positions of homologous genes were consistent with their order on the donor genomes (Figure 1). *G. hirsutum* (AADD) is allotetraploid, and there is a homoeologous relationship between the A and D subgenomes. Numerous studies have shown that *G. raimondii* is the donor to the D subgenome of allotetraploid cotton such as *G. hirsutum* [46,47], and *G. arboreum* is a close relative of A subgenome in *G. hirsutum*, a finding supported by the homologous relationships among the PHT1 genes in *Gossypium*. However, the homologous analysis revealed that *GaPHT1;7* in *G. arboreum* and *GrPHT1;8* and *GrPHT1;9* in *G. raimondii* had no homologous gene in *G. hirsutum*. The Blastp analysis based on the bidirectional best hit (BBH) method identified that the identity of *GH_D02G1054* in *G. hirsutum* to *GaPHT1;7* was 96.37%, and that to *GrPHT1;9* was 98.66%. However, this gene has an incomplete A0109 domain, and thus, cannot be considered a member of the *G. hirsutum* PHT1 family. The highest identity of *GrPHT1;8* to a gene in *G. hirsutum* was 86.02% (*GhPHT1;2-Dt*), indicating that *GrPHT1;8* does not have homologous genes in *G. hirsutum*. 

Duplicated genes can be retained for long periods of time and develop functional deviations [48]. In the phylogenetic tree, *GaPHT1;7* and *GrPHT1;9* were the only members of Group III, indicating their difference from other members. Compared with the other members, they had one fewer motif at each N-terminal and C-terminal (Figure 3A). A motif is the secondary structure of a protein molecule and the constituent unit of the domain, and the domain is an important part of protein function. A difference in motif might cause the protein to form a different domain, thus, changing the protein’s function [49]. Protein synthesis begins at the N-terminal. The C-terminal is important in determining the biological function of the protein. Thus, two genes—*GaPHT1;7* and *GrPHT1;9—*may be functionally differentiated from the other members of the PHT1 family.

Promoters known to be related to Pi absorption and transport include W-box elements in *BnPHT1;4* of *Brassica napus*, P1BS-like and PHO-like motifs in barley PHT1 promoters, P1BS elements in *A. thaliana*, rice and barley PHT1 promoters, and Myc-box in eggplant and tobacco PHT1 promoters [50,51,52]. The elements Myc-box (TTTCTTGT) and P1BS (GNATATNC) were not found in the cotton promoters (Supplementary Table S1). P1BS can interact with PHR1 transcription factors, but it is not a specific element that encodes a PHT1 gene. It has been reported that all dicotyledonous PHT1 promoters induced by arbuscular mycorrhizal fungi (AMF) contain Myc-box and P1BS elements. AMF help plants obtain nutrients, especially P. 

### 3.2. PHT1 Gene Responses to P Deficiency in Cotton

PHT1 is a high-affinity P transporter in plants and is responsible for maintaining P concentrations in plants in low-Pi environments. Accumulating evidence has revealed that the expression of PHT1 genes changes under low-Pi stress. In *A. thaliana*, *AtPHT1;8* and *AtPHT1;9* have been shown to be strongly induced in roots by P deficiency and to be involved in P transport from the roots to the aerial parts [14]. In rice, the expression of *OsPT2*, *OsPT4*, *OsPT8*, *OsPT9* and *OsPT10* was found to increase significantly under low-Pi induction [17,18,19,20]. In soybean, P deficiency alters the expression of *GmPT1*, *GmPT2* and *GmPT5* [23,24]. In *Solanaceae*, low-Pi stress has been shown to induce strong expression of *LePT1* and *LePT2* in tomato [26,53], and enhance the expression of *StPHT1;1* in potato leaf and root [54].

In our study, transcriptome data analysis showed that *GhPHT1;3-At* and *GhPHT1;3-Dt* in *G. hirsutum* were expressed in roots, stems, leaves and petals; these genes may be involved in long-distance Pi transport (Figure 6C). Studies have shown that P plays a vital role in cotton reproductive growth [5]. During reproductive growth, the proportion of P allocated to reproductive organs, such as flower buds, increases. At the boll-opening stage, the amount of P allocated to the reproductive organs peaks. The expression of *GhPHT1;4-At*, *GhPHT1;2-Dt* and *GhPHT1;6-Dt* predominantly occurs in reproductive organs, indicating that these genes might be involved in reproductive growth. 

In *G. arboreum* treated with low P, root expression of *GaPHT1;1*, *GaPHT1;3*, *GaPHT1;4*, *GaPHT1;5* and *GaPHT1;7* was significantly increased at 3 h relative to at 0 h (Figure 8A). Genes that were significantly upregulated by P starvation induction might be crucial genes. In *G. hirsutum* under low-Pi stress, the expression of *GhPHT1;3-At* and *GhPHT1;3-Dt* began to change early (within 1 to 3 h), and *GhPHT1;4-At*, *GhPHT1;5-At*, *GhPHT1;2-Dt*, *GhPHT1;4-Dt*, *GhPHT1;5-Dt*, and *GhPHT1;6-Dt* exhibited changes in expression after 6 h of stress (Figure 8B). 

RNA-seq analysis of *G. arboreum* revealed that *GaPHT1;4* and *GaPHT1;5* were highly expressed in the roots (Figure 6A); their homologous genes *GhPHT1;4-At* and *GhPHT1;5-At* in *G. hirsutum* were also highly expressed in the roots (Figure 6C). In *G. raimondii*, *GrPHT1;4* was highly expressed in the roots (Figure 6B); similarly, its homologous gene *GhPHT1;4-Dt* in *G. hirsutum* was highly expressed in the roots. In *G. hirsutum*, *GhPHT1;5-Dt* (D subgenome), homologous to *GhPHT1;5-At* (A subgenome), was highly expressed in the roots (Figure 6C). The qRT-PCR analysis showed that the expression of *GhPHT1;4-At*, *GhPHT1;5-At*, *GhPHT1;4-Dt* and *GhPHT1;5-Dt* in the roots was upregulated continuously under P starvation (Figure 8B). In summary, the above results suggest that the members of two pairs of homologous genes (*GhPHT1;4-At*, *GhPHT1;5-At*, *GhPHT1;4-Dt* and *GhPHT1;5-Dt*) are crucial genes for P uptake by roots in P starvation conditions. The findings of this study will accelerate the elucidation of the mechanisms of P absorption, transport and distribution and the development of cotton varieties with high P use efficiency.

## 4. Materials and Methods 

### 4.1. Genome-Wide Identification of PHT1 Genes in Three Gossypium Species

The whole genome [55] and protein [46] sequences of *G. arboreum*, *G. raimondii* and *G. hirsutum* were downloaded from Cotton Database Resources (https://www.cottongen.org/, accessed on 22 January 2019), and the PHT1 protein sequence of *A. thaliana* was downloaded from The Arabidopsis Information Resource (TAIR: http://www.arabidopsis.org/, accessed on 18 February 2019). The conserved domain of the PHT1 protein was analyzed by Pfam (http://pfam.xfam.org/, accessed on 8 June 2019). Then, the domain was searched in the whole cotton genome by HMMER3.3 (hmmer.org, accessed on 8 June 2019), and the possible PHT1 genes were extracted. Using ClustalW2 software, multiple sequences of the putative genes were aligned [56] to construct an accurate Hidden Markov Model (HMM) of PHT1 in cotton. Using HMM, possible PHT1 family genes were re-extracted from the cotton genome database. The extracted results were subjected to phylogenetic analysis with *A. thaliana* PHT1 genes, and the genes with similar sequences were subjected to a NCBI Conserved Domain search to determine the integrity of the domain.

### 4.2. Location and Structural Analysis of PHT1 Genes

MapChart [57] was used to locate PHT1 genes on chromosomes. The positions of exons and introns were predicted by GSDS2.0 software [58]. Conservative motif prediction was performed by MEME software [59], and the maximum number of motifs was set to 12. The maximum width of the logo was 50, and the minimum was 6. TBtools [60] was employed to produce the figures on gene structure, motif structure and the phylogenetic tree. PlantCARE [61] was for promoter analysis.

### 4.3. Sequence Alignment and Phylogenetic Tree Construction 

ClustalW2 [56] was used to compare the amino acid (aa) sequences of PHT1 family members. For pairwise parameters, the gap opening was set at 10.00, and the gap extension was set at 0.10; for multiple parameters, Gap Opening Penalty was set at 10.00, and Gap Extension Penalty was set at 0.20. MEGA X [62] software was applied to construct the phylogenetic tree by ML with the bootstrap method (number of bootstrap replications = 1000) and the Poisson model. The tree was visualized using iTOL [63].

### 4.4. Analysis of Synteny and Duplication Types

MCScanX [41] was used for synteny analysis with the following parameters—match score: 50; gap penalty: −1; match size: 5; e value: 1e-05; max gaps: 25. Duplication event analysis was performed with the results of the synteny analysis. The results were visualized by Circos [42]. The KaKs_Calculator [64] was used to calculate Ka and Ks. The syntenic data of PHT1 genes in *G. arboreum*, *G. raimondii* and *G. hirsutum* were extracted using Perl script. Blastp (2.7.1+), based on the BBH method, was used to identify homologous gene relationships among the three cotton species. The synteny among the three cotton species was calculated by MCScan (python version) software, and the running platform was jcvi.

### 4.5. P starvation Treatment

*G. hirsutum* acc. TM-1 and *G. arboreum* acc. Shixiya 1 were used for gene expression analysis. Cotton plants were hydroponically grown in Hoagland nutrient solution (with the Pi concentration set to 1 × 10^−3^ mol/L). For P deficiency treatment, the Pi concentration was set to 1 × 10^−5^ mol/L, with the remaining ingredients added according to the levels in Hoagland nutrient solution. The missing K^+^ was supplemented in the form of KCl. Pi-deficiency treatment was initiated at the first-true-leaf stage. Samples of leaves and roots were collected at 0, 1, 3, 6, 12 and 24 h after low-Pi treatment, frozen in liquid nitrogen and stored at −70 °C for later use. *G. hirsutum* acc. TM-1 high-throughput RNA-sequencing data from Zhang et al. [47] were analyzed for the expression patterns of the PHT1 genes in different tissues. Transcriptome data of *G. arboreum* were downloaded from NCBI BioProject PRJNA349094 [65], and transcriptome data of *G. raimondii* were downloaded from NCBI BioProject PRJNA507768 [66]. Gene expression was expressed as Fragments Per Kilobase per Million (FPKM), and heat maps were constructed using the R package pheatmap.

### 4.6. RNA Extraction and qRT-PCR

Total RNA was extracted by the Cetyltrimethylammonium bromide (CTAB) -acidic phenolic method described by Jiang and Zhang [67]. HiScript qRT SuperMix for qPCR (+gDNAwiper) (Vazyme Biotech Co., Ltd., Nanjing, China) was used to remove genomic DNA and reverse transcribe the RNA to synthesize first-strand cDNA. The primers (Supplementary Table S4) for qRT-PCR were designed by Primer 5.0 (http://www.premierbiosoft.com/, accessed on 20 July 2019). The amplified product size was between 100 and 300 bp, and the annealing temperature was between 58 and 60 °C. The specificity of the primers was detected using the primer-blast tool (https://www.ncbi.nlm.nih.gov/tools/primer-blast/, accessed on 21 July 2019) of NCBI. The Histone3 (AF024716) gene (forward primer and reverse primer sequences 5’GAAGCCTCATCGATACCGTC-3’ and 5’-CTACCACTACCATCATGG-3’, respectively) was used as an internal reference gene. qRT-PCR amplification was performed with a LightCycler ^®^480 Instrument II (Roche). The cycling conditions were as follows: 95 °C for 30 s, followed by 40 cycles of 95 °C for 10 s and 60 °C for 30 s. The melting curve was analyzed using LightCycler^®^ 480 software 1.5. Each experiment was performed with three biological replicates and three technical replicates. SYBR Green I was utilized in qRT-PCR procedure [68]. A delta-delta Ct method was used for the estimation of relative transcription levels according to Livak and Schmittgen [69]. Analysis of variance [45] was performed with SPSS (https://www.ibm.com/support/ pages/node/417815, accessed on 3 August 2019).

## Figures and Tables

**Table 1:** PHT1 family genes in *G. arboreum*, *G. raimondii* and *G. hirsutum*.

Species	Name	Gene ID	Number of Amino Acid	Molecular Weight (kDa)	Isoelectric Point
*G. arboreum*	*GaPHT1;1*	108475583	531	58.14	9.06
	*GaPHT1;1.1*	108475338	529	58.08	8.90
	*GaPHT1;1.2*	108475339	529	58.10	8.69
	*GaPHT1;2*	108477504	527	57.84	9.12
	*GaPHT1;3*	108462460	540	59.15	8.79
	*GaPHT1;4*	108454927	537	58.51	8.85
	*GaPHT1;5*	108472823	533	58.07	8.07
	*GaPHT1;6*	108488770	533	58.10	8.66
	*GaPHT1;7*	108454997	524	58.13	9.05
*G. hirsutum*	*GhPHT1;1-At*	GH_A02G0225	529	58.27	8.69
	*GhPHT1;1.1-At*	GH_A02G0226	529	58.20	8.68
	*GhPHT1;1.2-At*	GH_A02G0227	531	58.29	8.99
	*GhPHT1;2-At*	GH_A07G1973	527	57.86	9.24
	*GhPHT1;3-At*	GH_A07G2404	540	59.14	8.78
	*GhPHT1;4-At*	GH_A09G0772	537	58.56	8.73
	*GhPHT1;5-At*	GH_A10G1372	533	58.10	8.07
	*GhPHT1;6-At*	GH_A11G1359	533	58.14	8.66
	*GhPHT1;1-Dt*	GH_D02G0244	529	58.16	8.99
	*GhPHT1;1.1-Dt*	GH_D02G0245	529	58.21	8.78
	*GhPHT1;1.2-Dt*	GH_D02G0246	531	58.15	8.99
	*GhPHT1;2-Dt*	GH_D07G1977	525	57.74	9.29
	*GhPHT1;3-Dt*	GH_D07G2348	540	59.23	8.67
	*GhPHT1;4-Dt*	GH_D09G0713	537	58.50	8.94
	*GhPHT1;5-Dt*	GH_D10G1509	533	58.11	8.07
	*GhPHT1;6-Dt*	GH_D11G1391	533	58.10	8.78
*G. raimondii*	*GrPHT1;1*	105796073	529	58.22	8.98
	*GrPHT1;2*	105801425	525	57.77	9.25
	*GrPHT1;3*	105767689	540	59.19	8.79
	*GrPHT1;4*	105798813	537	58.54	8.98
	*GrPHT1;5*	105774918	533	58.11	8.08
	*GrPHT1;6*	105803201	533	58.16	8.66
	*GrPHT1;8*	105796728	513	56.74	9.08
	*GrPHT1;9*	105793869	524	58.19	9.17

**Table 2:** Comparison of PHT1 homologous genes substitution rate in *Gossypium*.

Homoeologous Genes	Ka	Ks	Ka/Ks
*GaPHT1;1*-*GhPHT1;1-At*	0.0367	0.1208	0.3039
*GaPHT1;1.1-GhPHT1;1.1-At*	0.0247	0.0677	0.3646
*GaPHT1;1.2-GhPHT1;1.2-At*	0.0308	0.1214	0.2541
*GaPHT1;2*-*GhPHT1;2-At*	0.0032	0.0111	0.2851
*GaPHT1;3*-*GhPHT1;3-At*	0.0026	0.0094	0.2798
*GaPHT1;4*-*GhPHT1;4-At*	0.0066	0.0179	0.3662
*GaPHT1;5*-*GhPHT1;5-At*	0.0009	0.0103	0.0867
*GaPHT1;6*-*GhPHT1;6-At*	0.0017	0.0046	0.3665
*GrPHT1;1*-*GhPHT1;1-Dt*	0.0076	0.0357	0.2130
*GrPHT1;2*-*GhPHT1;2-Dt*	0.0025	0.0029	0.8520
*GrPHT1;3*-*GhPHT1;3-Dt*	0.0024	0.0053	0.4559
*GrPHT1;4*-*GhPHT1;4-Dt*	1.0542	0.8497	1.2407
*GrPHT1;5*-*GhPHT1;5-Dt*	0.0017	0.0146	0.1149
*GrPHT1;6*-*GhPHT1;6-Dt*	0.0008	0.0029	0.2800

## Supplementary Materials

Supplementary materials can be found at https://www.mdpi.com/1422-0067/21/14/4905/s1. Figure S1. Phylogenetic tree based on *A. thaliana* PHT1 protein sequences and cotton PHT1 candidate protein sequences. Figure S2. The phylogenetic tree of PHT1 genes in *Gossypium*, *O. sativa* and *A. thaliana*. Table S1. Cis-acting element of known Pi transporter predicted in *Gossypium*. Table S2. Results of 2^(-∆Ct) in qRT-PCR of *GaPHT1* and *GhPHT1* (in leaf). Table S3. Results of 2^(-∆Ct) in qRT-PCR of *GaPHT1* and *GhPHT1* (in root). Table S4. Primer sequences used for relative expression analysis of Pi transporter genes from cotton.

## Abbreviations

P	phosphorus
Pi	phosphate
PHT	phosphate transporter
MFS	Major Facilitator Superfamily
aa	amino acid
pIs	isoelectric points
ML	Maximum Likelihood
GSDS	Gene Structure Display Server
BBH	Bidirectional Best Hit
AMF	*arbuscular mycorrhizal* fungi
HMM	Hidden Markov Model
qRT-PCR	quantitative real time PCR
MEME	Multiple Em for Motif Elicitation
Ka	Non-synonymous substitution
Ks	Synonymous substitution
