# Supplementary material for: Genome-Wide Identification and Expression Profile Analysis of the PHT1 Gene Family in Gossypium hirsutum and Its Two Close Relatives of Subgenome Donor Species
Source: Int J Mol Sci. 2020 Jul 11;21(14):4905. doi: 10.3390/ijms21144905 (PMC7404403; doi:10.3390/ijms21144905)
Supplement: Supplementary file 1 [file ijms-21-04905-s001.zip › supplementary/supplementary figures.pdf]

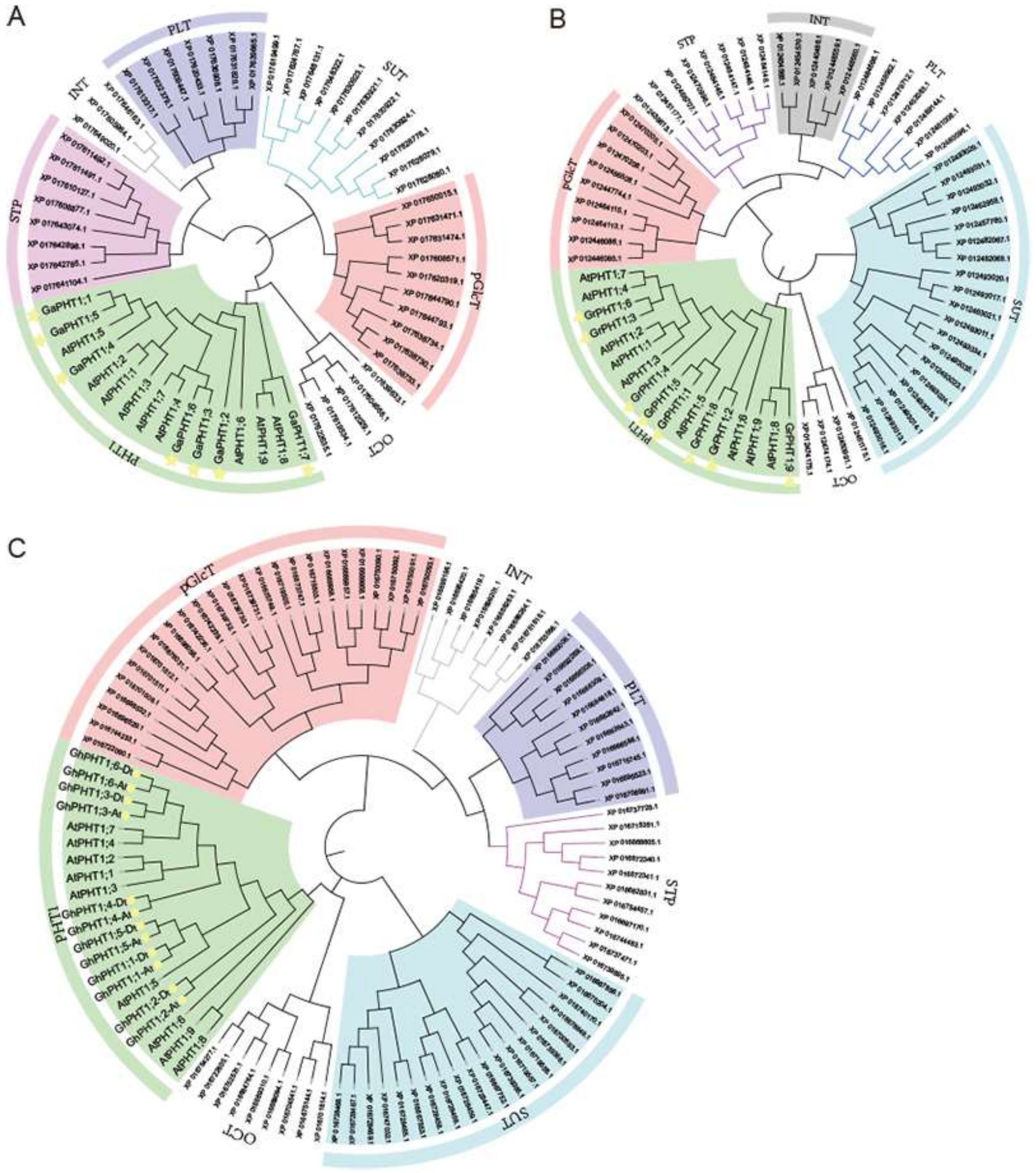

Figure S1. Phylogenetic tree based on *A. thaliana* PHT1 protein sequences and cotton PHT1 candidate protein sequences. In the protein label notes, the prefix "Ga", "Gr", "Gh" and "At" stands for *G. arboreum*, *G. raimondii*, *G. hirsutum* and *A. thaliana*, respectively. The candidate genes include seven main groups, namely, PHT1, STP, INT, PLT, SUT, pGlcT and OCT. PHT1 represents inorganic phosphate transporter, STP represents sugar transport protein, INT represents inositol transporter, PLT represents polyol transporter, SUT represents sugar transporter ERD6-like, pGlcT represents plastidic glucose transporter and OCT represents organic cation/carnitine transporter. A, *G. arboreum.* B, *G. raimondii*. C, *G. hirsutum.*

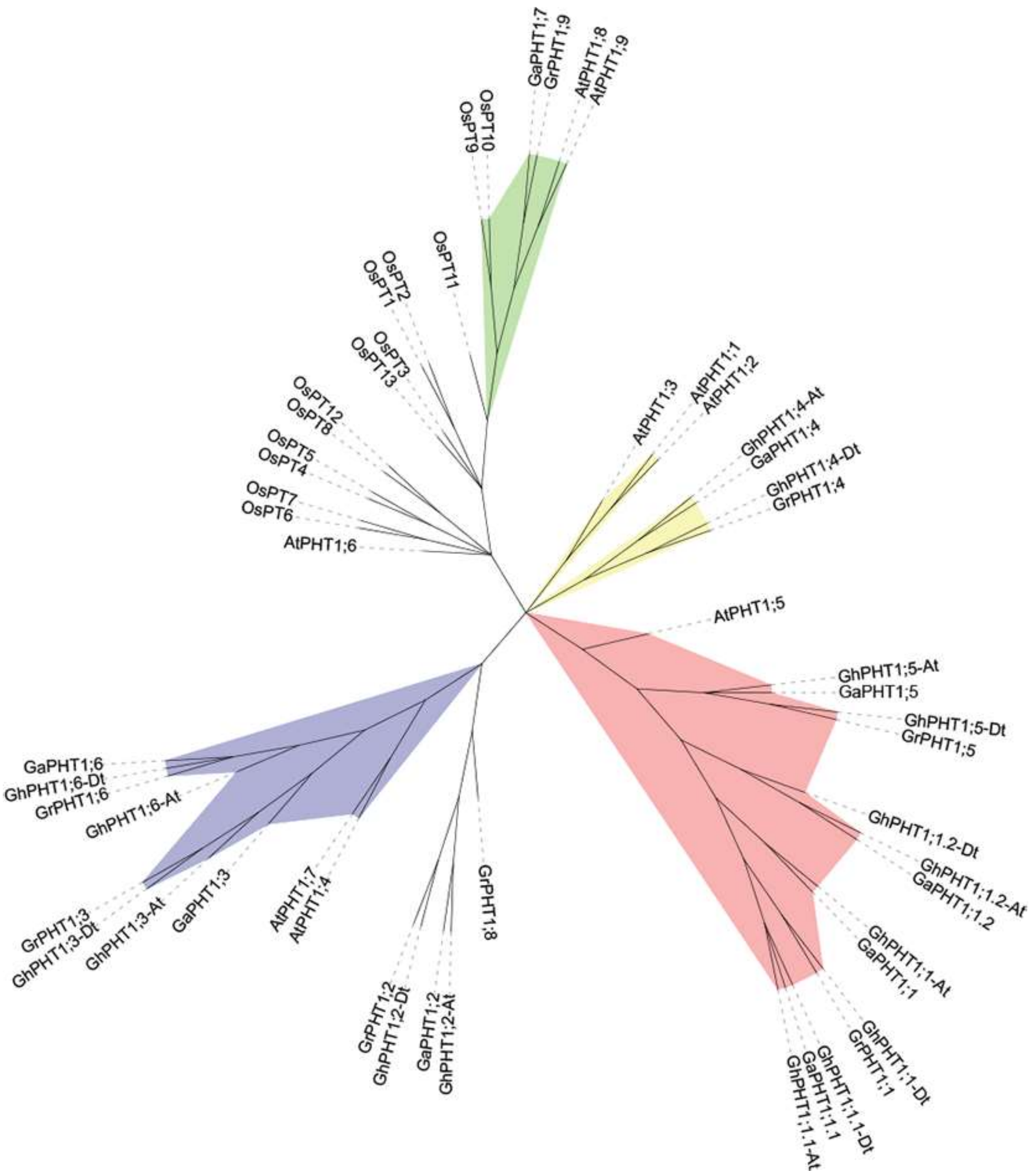

Figure S2. The phylogenetic tree of PHT1 genes in *Gossypium*, *O. sativa* and *A. thaliana*. Ga-, Gr- and Gh- represent PHT1 genes from *G. arboreum*, *G. raimondii* and *G. hirsutum*, respectively. Os- represent PHT1 genes from rice, At- represent PHT1 genes from *A. thaliana*. PHT1 genes originating from the same ancestor are marked with the same color.
