# Supplementary material for: Genome-Wide Identification and Expression Profile Analysis of the PHT1 Gene Family in Gossypium hirsutum and Its Two Close Relatives of Subgenome Donor Species
Source: Int J Mol Sci. 2020 Jul 11;21(14):4905. doi: 10.3390/ijms21144905 (PMC7404403; doi:10.3390/ijms21144905)
Supplement: Supplementary file 1 [file ijms-21-04905-s001.zip › supplementary/supplementary tables.pdf]

Table S1. Cis-acting element of known phosphate transporter predicted in *Gossypium.*

Name	Domain	Sequence	Site
GrPHT1;2	W box	TTGACC	1573
GhPHT1;3-At	TATA-box	TATATTTATATTT	170
GrPHT1;3	TATA-box	TATATTTATATTT	1547
GhPHT1;3-Dt	TATA-box	TATATTTATATTT	189
GaPHT1;4	O2-site	GTTGACGTGA	122
GhPHT1;4-At	W box	TTGACC	891
GhPHT1;4-At	W box	TTGACC	1102
GrPHT1;4	O2-site	GTTGACGTGA	1944
GrPHT1;4	O2-site	GATGA(C/T)(A/G)TG(A/G)	698
GhPHT1;4-Dt	O2-site	GTTGACGTGA	312
GhPHT1;4-Dt	W box	TTGACC	1479
GaPHT1;5	W box	TTGACC	1397
GhPHT1;5-At	W box	TTGACC	599
GrPHT1;9	O2-site	GATGATGTGG	1106

Note: The upstream 2000bp sequences before the initiation codon of PHT1 genes were selected as the promoter region to predict the possible cis-acting elements. The sites of these elements were based on analysis of the sequence data in this region.

Table S2. Results of $2^{-\Delta Ct}$ in qRT-PCR of *GaPHT1* and *GhPHT1* (in leaf).

Gene name	0 h	1 h	3 h	6 h	12 h	24 h
GaPHT1;1	-	-	-	-	-	-
GaPHT1;2	-	-	-	-	-	-
GaPHT1;3	0.004047	0.005982	0.011253	0.001761	0.002587	0.004112
GaPHT1;4	0.008358	0.034997	0.002975	0.001152	0.001492	0.003061
GaPHT1;5	-	-	-	-	-	-
GaPHT1;6	-	-	-	-	-	-
GaPHT1;7	0.000721	0.001020	0.001723	0.000894	0.000901	0.001318
GhPHT1;1-At	-	-	-	-	-	-
GhPHT1;2-At	-	-	-	-	-	-
GhPHT1;3-At	0.003661	0.004287	0.004326	0.003212	0.006782	0.002644
GhPHT1;4-At	-	-	0.000215	0.000186	0.001525	0.000380
GhPHT1;5-At	-	-	-	-	-	0.000195
GhPHT1;6-At	-	-	-	-	-	-
GhPHT1;1-Dt	-	-	-	-	-	-
GhPHT1;2-Dt	-	-	-	-	-	0.000113
GhPHT1;3-Dt	0.004536	0.007978	0.007861	0.005512	0.019122	0.005549
GhPHT1;4-Dt	-	-	-	-	0.000545	0.000222
GhPHT1;5-Dt	-	-	-	-	-	0.000124
GhPHT1;6-Dt	-	-	-	-	-	-

Note: “-” represents that PCR products could not be detected with gene-specific primers. The value was from the average of three replicates. 0 h, 1 h, 3 h, 6 h, 12 h and 24 h represent the time with low-Pi treatment.

Table S3. Results of $2^{-\Delta Ct}$ in qRT-PCR of *GaPHT1* and *GhPHT1* (in root).

Gene name	0 h	1 h	3 h	6 h	12 h	24 h
GaPHT1;1	0.003052	0.004029	0.004703	0.001021	0.002641	0.000687
GaPHT1;2	-	-	-	-	-	-
GaPHT1;3	0.000727	0.009220	0.019690	0.008925	0.006853	0.002654
GaPHT1;4	0.085001	0.068217	0.561700	0.160246	0.199981	0.127943
GaPHT1;5	0.002703	0.006634	0.026369	0.008129	0.006413	0.002261
GaPHT1;6	-	-	-	-	-	-
GaPHT1;7	0.001099	0.004059	0.003430	0.000546	0.001674	0.000711
GhPHT1;1-At	-	-	-	-	-	-
GhPHT1;2-At	-	-	-	-	-	-
GhPHT1;3-At	0.000704	0.001566	0.004187	0.002313	0.001854	0.001257
GhPHT1;4-At	0.084078	0.055461	0.123278	0.231033	0.326822	0.542399
GhPHT1;5-At	0.000977	0.001095	0.009238	0.007701	0.022624	0.021164
GhPHT1;6-At	-	-	-	-	-	-
GhPHT1;1-Dt	-	-	-	-	-	-
GhPHT1;2-Dt	0.000289	0.000065	0.000105	0.000388	0.001257	0.000662
GhPHT1;3-Dt	0.001245	0.002466	0.005777	0.002314	0.002318	0.003997
GhPHT1;4-Dt	0.032007	0.018732	0.031733	0.088686	0.193952	0.326525
GhPHT1;5-Dt	0.000890	0.001125	0.003496	0.006868	0.010131	0.014852
GhPHT1;6-Dt	0.000088	0.000121	0.000131	0.000156	0.000437	0.000908

Note: "-" represents that PCR products could not be detected with specific primers. The value was from the average of three repeats. 0 h, 1 h, 3 h, 6 h, 12 h and 24 h represent the time with low-Pi treatment.

Table S4. Primer sequences used for relative expression analysis of phosphate transporter genes from cotton.

Gene Name	Forward primer (5′–3′)	Forward primer (5′–3′)
GaPHT1;3	CGGACCTAATGCCACCACTT	CCCGAATGCGCCAACAATAG
GaPHT1;2	CGATTCTGGCTCGGTTTTGG	GGTGAAGGGTATTGGGCCTT
GaPHT1;5	AAGATTCGACTGCGCCTGAA	TTTGCGTTCCTCGCAACAAG
GaPHT1;1	GCTGCACAAAGCACACATGA	CAGTTGACTGCACCAAGCAC
GaPHT1;6	TAAGAGCCCCGGATCATTGC	AAGGGTTGCCATCACTGCTT
GaPHT1;7	GTACGGGCTGTCATTAGGCA	GGCTGCTGACAAAGGGTAGT
GaPHT1;4	ACGGTGTTGCTTTAGTCGGT	GCCGAAAGAGGGTAGTCACC
GhPHT1;1-At	CGAGTTGCCAAGGCTCAAAC	GTGTTCGATGAAAGCGACCG
GhPHT1;2-At	TTTGCCTCTTGCAAAGCCTG	TCACCGAGCCAACCAAAGAA
GhPHT1;3-At	AAGAATGCAAAGCAGGCAGC	TTCCAAGCAAGTGAAGCCCA
GhPHT1;4-At	GTGTTCGCAATGCAAGGGTT	GCAACAAGGGCTGTAAACCG
GhPHT1;5-At	AAGATTCGACTGCGCCTGAA	TTTGCGTTCCTCGCAACAAG
GhPHT1;6-At	AACCACCACTTGGTTCTTGC	CCAGTGCAAACATGAACACC
GhPHT1;1-Dt	CACCGTACCGGAAGCTGATT	TGCCAAGCAAGTGAACTCCA
GhPHT1;2-Dt	CATGGGCTTCACTTGCTTGG	GCGTTCATGGTTTTCGCCTT
GhPHT1;3-Dt	TGGACCCACAAGGACAATCG	TTGCTGGGAAAATCTCGGCT
GhPHT1;4-Dt	TGGTTCACGGTGGCATTGAT	GGGAAGATCTCAGCTGGCAC
GhPHT1;5-Dt	CCAAAAGCCGCAACCATGAA	CAGTATCCAGGGATCGTGCC
GhPHT1;6-Dt	GCCGAAAGAAGGTCTACGGT	CAAAGGGTTGCCATCACTGC
